# Supplementary material for: The Role of Virulence Factors in Neonatal Sepsis Caused by Enterobacterales: A Systematic Review
Source: Int J Mol Sci. 2022 Oct 8;23(19):11930. doi: 10.3390/ijms231911930 (PMC9569801; doi:10.3390/ijms231911930)
Supplement: Supplementary file 1 [file ijms-23-11930-s001.zip › ijms-1935376-supplementary.pdf]

International Journal of *Molecular Sciences*

MDPI

The Role of Virulence Factors in Neonatal Sepsis Caused by Enterobacterales: A Systematic Review

Lucia Barcellini [1,*], Giulia Ricci [1], Ilia Bresesti [2], Aurora Piazza [3], Francesco Comandatore [4], Mike Sharland [5], Gian Vincenzo Zuccotti [1] and Laura Folgori [1]

Supplementary Material

Search strategy

Ovid MEDLINE(R) <1946 to March Week 2 2022>

1 sepsis.mp. or exp Sepsis/

2 septi*.mp.

3 exp Bacteremia/ or septicaemi*.mp.

4 ((blood?stream or (blood adj1 stream)) adj1 infect*).mp. [mp=title, abstract, original title, name of substance word, subject heading word, floating sub-heading word, keyword heading word, organism supplementary concept word, protocol supplementary concept word, rare disease supplementary concept word, unique identifier, synonyms]

5 1 or 2 or 3 or 4

6 neonate.mp. or exp Infant, Newborn/

7 infant.mp. or exp Infant/

8 newborn.mp.

9 6 or 7 or 8

10 *Escherichia coli/ or exp *Virulence/ or *Bacterial Proteins/ or exp *Virulence Factors/

11 *Escherichia coli Infections/ or *Escherichia coli/ or *Bacterial Proteins/ or exp *Virulence Factors/ or *Genes, Bacterial/ or exp *Virulence/

12 *Klebsiella pneumoniae/ or exp *Virulence Factors/ or *Escherichia coli/ or *Klebsiella Infections/ or *Bacteria/ or exp *Virulence/ or *Escherichia coli Infections/

13 exp *Virulence Factors/ or exp *Virulence/

14 adherence.mp

15 *Bacterial Proteins/ or *Bacteria/ or *Escherichia coli/ or *Escherichia coli Proteins/

16 *Gastrointestinal Microbiome/ or *Bacterial Proteins/ or *Bacteria/ or *Escherichia coli/ or *Escherichia coli Proteins/

17 capsule.mp. or *Capsules/

18 cell invasion.mp.

19 *Iron/ or iron metabolism.mp

20 motility.mp.

21 chemotaxis.mp.

22 pumps.mp.

23 *Gene Expression Regulation, Bacterial/ or exp *Bacterial Proteins/ or exp *Virulence Factors/ or *Type III Secretion Systems/

24 toxins.mp. or exp *Bacterial Toxins/ or *Shiga Toxins/

25 anti-aggregation.mp.

26 exp *Adhesins, Escherichia coli/ or exp *Adhesins, Bacterial/

27 exp *Fimbriae Proteins/ or exp *Fimbriae, Bacterial/

28 *Enteropathogenic Escherichia coli/ or *Escherichia coli/ or *Escherichia coli O157/ or *Bacterial Outer Membrane Proteins/ or exp *Adhesins, Bacterial/ or *Shiga-Toxigenic Escherichia coli/ or *Escherichia coli Proteins/ or *Escherichia coli Infections/

29 *Zinc/ or *Membrane Proteins/ or *Metalloproteases/ or *Bacterial Proteins/

30 exp *Virulence Factors/ or exp *Klebsiella pneumoniae/ or *Allantoin/ or *Klebsiella Infections/
31 transcription factors.mp.
32 *Transcription Factors/ or *Gene Expression Regulation, Bacterial/ or *Escherichia coli/ or exp *Bacterial Proteins/ or *Promoter Regions, Genetic/ or bacterial survival promoters.mp.
33 lipopolysaccharide.mp.
34 arylsulfatase.mp. or Arylsulfatases/
35 exp *Bacterial Outer Membrane Proteins/ or *Membrane Proteins/ or *Porins/ or *Escherichia coli/
36 invasion protein A.mp.
37 siderophores.mp. or Siderophores/
38 *Bacterial Outer Membrane Proteins/ or *Bacterial Proteins/ or *Hemin/ or *Membrane Proteins/
39 enterobactin.mp. or Enterobactin/
40 exp *Virulence Factors/ or *Klebsiella pneumoniae/ or *Escherichia coli/ or *Bacterial Proteins/ or *Escherichia coli Infections/ or exp *Siderophores/ or *Klebsiella Infections/
41 *Klebsiella pneumoniae/ or exp *Virulence Factors/ or exp *Siderophores/ or *Escherichia coli Infections/ or *Bacterial Proteins/ or *Enterobactin/ or *Escherichia coli/ or *Klebsiella Infections/
42 exp *Virulence Factors/ or *Bacterial Proteins/ or exp *Siderophores/ or *Klebsiella Infections/ or exp *Virulence/ or *Escherichia coli Infections/ or *Escherichia coli/ or *Klebsiella pneumoniae/
43 *Bacterial Proteins/ or haemoglobin transport protein.mp.
44 flagella.mp. or Flagella/
45 exp *Virulence Factors/ or *Type IV Secretion Systems/ or *Bacterial Proteins/ or *Type III Secretion Systems/ or exp *Bacteria/ or exp *Bacterial Outer Membrane Proteins/ or *Bacterial Secretion Systems/
46 *Gene Expression Regulation, Bacterial/ or *Membrane Proteins/ or *Bacterial Proteins/ or *Escherichia coli Proteins/ or *Bacterial Outer Membrane Proteins/ or *Bacterial Secretion Systems/
47 *Type III Secretion Systems/ or *Escherichia coli Proteins/ or *Bacterial Proteins/ or *Antigens, Bacterial/ or *Gene Expression Regulation, Bacterial/ or *Salmonella typhimurium/
48 *Gene Expression Regulation, Bacterial/ or *Escherichia coli/ or *Type VI Secretion Systems/ or *Bacterial Secretion Systems/ or *Bacterial Proteins/ or *Gram-Negative Bacteria/ or exp *Virulence Factors/
49 *Escherichia coli Infections/ or *Klebsiella pneumoniae/ or *Escherichia coli Proteins/ or *Escherichia coli/
50 *Escherichia coli Proteins/ or *Salmonella typhimurium/ or *Type III Secretion Systems/ or *Bacterial Proteins/ or *Bacterial Secretion Systems/ or exp *Virulence Factors/ or *Host-Pathogen Interactions/
51 *Virulence Factors/ or Bacterial Proteins/ or *Hemolysin Proteins/ or *Escherichia coli/ or *Bacterial Toxins/
52 *Bacterial Proteins/ or aap.mp.
53 *Escherichia coli/ or *Adhesins, Escherichia coli/
54 Escherichia coli/ or dra.mp.
55 fde.mp.
56 agg.mp.
57 bcf.mp.
58 csg.mp.
59 daa.mp.
60 fim.mp.

61 foc.mp.
62 *Fimbriae, Bacterial/ or lpf.mp.
63 mrk.mp.
64 paa.mp.
65 pap.mp. or *Phosphoadenosine Phosphosulfate/
66 sfa.mp.
67 eae.mp.
68 *Escherichia coli/ or tir.mp. or *Escherichia coli Proteins/
69 stc.mp.
70 map.mp.
71 mgt.mp.
72 tcp.mp.
73 cps.mp.
74 gal.mp.
75 glf.mp.
76 gnd.mp. or *Genes, Bacterial/
77 gtr.mp.
78 kfo.mp.
79 kps.mp.
80 man.mp.
81 rcs.mp.
82 rmp.mp.
83 *Salmonella typhimurium/ or *Escherichia coli Proteins/ or *Bacterial Proteins/ or *Escherichia coli/ or *Gene Expression Regulation, Bacterial/
84 wca.mp.
85 *Bacterial Outer Membrane Proteins/ or *Bacterial Capsules/ or *Escherichia coli Proteins/ or *Klebsiella pneumoniae/ or wza.mp. or *Bacterial Proteins/ 172468
86 *Klebsiella Infections/ or *Klebsiella pneumoniae/ or *Bacterial Capsules/ or *Bacterial Proteins/ or wzi.mp.
87 wzm.mp.
88 wzt.mp.
89 lpx.mp.
90 exp *Lipopolysaccharides/ or waa.mp.
91 wbb.mp.
92 asl.mp.
93 *Escherichia coli/ or *Bacterial Outer Membrane Proteins/ or *Escherichia coli Proteins/ or ompA.mp. or *Bacterial Proteins/
94 *Escherichia coli Infections/ or *Escherichia coli/ or *Virulence Factors/ or ibeA.mp. or *Virulence/ or *Escherichia coli Proteins/ or *Membrane Proteins/
95 chu.mp.
96 ent.mp.
97 fep.mp.
98 fes.mp.
99 fyu.mp.
100 irp.mp.
101 ybt.mp.
102 iro.mp.
103 iuc.mp.
104 iut.mp.
105 shu.mp.
106 che.mp.
107 mot.mp.
108 flg.mp.
109 flh.mp.

110 fli.mp.
111 acr.mp.
112 *Virulence Factors/ or *Bacterial Proteins/ or *Bacterial Toxins/ or hly.mp. or *Escherichia coli Infections/
113 exe.mp.
114 gsp.mp.
115 ces.mp.
116 esc.mp.
117 tssH.mp.
118 *Bacterial Secretion Systems/ or *Bacterial Proteins/ or *Type IV Secretion Systems/ or *Escherichia coli/ or *Escherichia coli Proteins/ or *Membrane Proteins/ or tssL.mp. or *Type VI Secretion Systems/
119 *Vasoactive Intestinal Peptide/ or vip.mp.
120 icmF.mp.
121 tss.mp. 5637
122 clb.mp.
123 esp.mp.
124 hly.mp.
125 10 or 11 or 12 or 13 or 14 or 15 or 16 or 17 or 18 or 19 or 20 or 21 or 22 or 23 or 24 or 25 or 26 or 27 or 28 or 29 or 30 or 31 or 32 or 33 or 34 or 35 or 36 or 37 or 38 or 39 or 40 or 41 or 42 or 43 or 44 or 45 or 46 or 47 or 48 or 49 or 50 or 51 or 52 or 53 or 54 or 55 or 56 or 57 or 58 or 59 or 60 or 61 or 62 or 63 or 64 or 65 or 66 or 67 or 68 or 69 or 70 or 71 or 72 or 73 or 74 or 75 or 76 or 77 or 78 or 79 or 80 or 81 or 82 or 83 or 84 or 85 or 86 or 87 or 88 or 89 or 90 or 91 or 92 or 93 or 94 or 95 or 96 or 97 or 98 or 99 or 100 or 101 or 102 or 103 or 104 or 105 or 106 or 107 or 108 or 109 or 110 or 111 or 112 or 113 or 114 or 115 or 116 or 117 or 118 or 119 or 120 or 121 or 122 or 123 or 124
126 Gram negative bacteria.mp. or *Gram-Negative Bacteria/
127 *Enterobacteriaceae/ or Enterobacterales.mp.
128 *Enterobacteriaceae/
129 *Escherichia coli/
130 *Klebsiella/
131 *Enterobacter/
132 *Proteus/
133 *Serratia/
134 126 or 127 or 128 or 129 or 130 or 131 or 132 or 133
135 5 and 9 and 125 and 134
136 limit 135 to humans

Embase (Embase 1974 to 2022 Week 12)

(('sepsis'/exp OR sepsis) OR bacteremia OR 'septicemia' OR 'bloodstream infection') AND ('newborn' OR 'infant') AND ('virulence factor' OR 'virulence gene' OR virulome OR 'virulence' OR 'adherence' OR 'bacterial metabolism' OR 'capsule' OR 'cell invasion' OR 'iron metabolism' OR 'motility' OR 'chemotaxis' OR 'pump' OR (secretion AND system AND factor) OR 'toxin' OR 'anti aggregation' OR 'adhesin' OR 'fimbria' OR 'intimin' OR 'zinc metalloprotease' OR (allantoin AND metabolism) OR (bacterial AND survival AND promoters) OR 'lipopolysaccharide' OR arylsulfatase OR 'outer membrane protein a' OR (invasion AND protein AND a) OR 'siderophore' OR (hemin AND uptake) OR enterobactin OR 'yersiniabactin' OR 'salmochelin' OR 'aerobactin' OR (haemoglobin AND transport AND protein) OR (haemoglobin AND transport AND receptor) OR 'flagellum' OR 'type i secretion system' OR 'type ii secretion system' OR 'type iii secretion system' OR t6ss OR 'type vi secretion system' OR 'colibactin' OR (t3ss AND effector) OR 'hemolysin a' OR 'aap gene' OR 'afa gene' OR 'dra gene' OR fde OR 'agg gene' OR 'bcfc gene' OR 'csga gene' OR 'csgd gene' OR 'csgb gene' OR (daa AND gene) OR 'fim gene' OR 'foc gene' OR 'lpfa gene' OR (mrk AND gene) OR 'paa gene' OR 'pap gene' OR 'sfa gene' OR (yag AND ecp) OR

ykgecp OR (ykg AND ecp) OR 'eae gene' OR 'tir gene' OR (stc AND gene) OR 'allantoin' OR ('allr s' AND gene) OR 'gene mapping' OR 'mgtc gene' OR 'tcp gene' OR 'cps1 gene' OR 'gal gene' OR (glf AND gene) OR 'gnd gene' OR (gtr AND gene) OR (kfo AND gene) OR (kps AND gene) OR (man AND gene) OR (rcs AND gene) OR (rmp AND gene) OR 'ugd gene' OR 'wcag gene' OR 'wza gene' OR 'wzi gene' OR 'wzm gene' OR wzt OR lpx OR waa OR wbb OR 'asl gene' OR 'ompa gene' OR 'ibea gene' OR 'ent gene' OR (chu AND gene) OR (fep AND gene) OR 'fes gene' OR 'fyua gene' OR 'irp1 gene' OR irp OR 'irp2 gene' OR 'ybta gene' OR 'ybts gene' OR (iro AND gene) OR iuc OR 'iuta gene' OR (shu AND gene) OR (che AND gene) OR (mot AND gene) OR 'flg gene' OR (flh AND gene) OR (fli AND gene) OR 'acr gene' OR 'hly gene' OR (exe AND gene) OR (gsp AND gene) OR (ces AND gene) OR (esc AND gene) OR clpv OR tssh OR dotu OR tssl OR hsib1 OR 'vip gene' OR icmf OR tss OR (clb AND gene) OR 'esp gene') AND ('gram negative bacterium' OR 'enterobacteriaceae' OR 'enterobacterales' OR 'klebsiella' OR (escherichia AND coli) OR 'proteus' OR 'serratia')

COCHRANE LIBRARY (Issue 3 of 12, March 2022

ID	Search	Hits
#1	(sepsis):ti,ab,kw (Word variations have been searched)	
#2	MeSH descriptor: [Sepsis] explode all trees	
#3	Bacteremia	
#4	MeSH descriptor: [Bacteremia] explode all trees	
#5	septicaemia	
#6	bloodstream infection	
#7	#1 OR #2 OR #3 OR #4 OR #5 OR #6	
#8	MeSH descriptor: [Infant, Newborn] explode all trees	
#9	infant	
#10	#8 OR #9	
#11	MeSH descriptor: [Virulence Factors] explode all trees	
#12	virulence gene	
#13	virulence	
#14	virulome	
#15	adherence	
#16	bacterial metabolism	
#17	capsule	
#18	cell invasion	
#19	iron metabolism	
#20	motility	
#21	chemotaxis	
#22	pumps	
#23	secretion system factors	
#24	Toxins	
#25	anti-aggregation	
#26	adhesin	
#27	fimbriae	
#28	intimin	
#29	zinc metalloprotease	
#30	allantoin metabolism	
#31	transcription factors	
#32	bacterial survival promoters	
#33	capsule	
#34	lipopolysaccharide	

#35	arylsulfatase	
#36	outer membrane protein A	
#37	invasion protein A	
#38	siderophores	
#39	hemin uptake	
#40	enterobactin	
#41	yersiniabactin	
#42	salmochelin	
#43	aerobactin	
#44	haemoglobin transport protein	
#45	haemoglobin transport receptor	
#46	chemotaxis	527
#47	flagella	24
#48	pumps	1826
#49	type I secretion system protein	
#50	type II secretion system protein	
#51	type III secretion system protein	
#52	type VI secretion system protein	
#53	colibactin	
#54	T3SS effector	
#55	hemolysin A	
#56	aap gene	
#57	afa	
#58	dra gene	
#59	fde	
#60	agg	
#61	bcf	
#62	csg	
#63	daa	
#64	fim	
#65	foc	
#66	lpf	
#67	mrk	
#68	paa	
#69	pap	
#70	sfa	
#71	yag	
#72	ecp	
#73	ykg	
#74	eae	
#75	tir	
#76	stc	
#77	all	
#78	map	
#79	mgt	
#80	tcp	
#81	cps	
#82	gal	
#83	glf	
#84	gnd	
#85	gtr	
#86	kfo	
#87	kps	
#88	man	

#89 rcs
#90 rmp
#91 ugd
#92 wca
#93 wza
#94 wzi
#95 wzm
#96 wzt
#97 lpx
#98 waa
#99 wbb
#100 asl
#101 ompA
#102 ibeA
#103 chu
#104 ent
#105 fep
#106 fes
#107 fyu
#108 irp
#109 ybt
#110 iro
#111 iuc
#112 iut
#113 shu
#114 mot
#115 che
#116 flg
#117 flh
#118 fli
#119 acr
#120 hly
#121 exe
#122 gsp
#123 ces
#124 esc
#125 clpV
#126 tssH
#127 dotU
#128 tssL
#129 hsiB1
#130 vip
#131 icmFtss
#132 tss
#133 esp
#134 #12 OR #13 OR #14 OR #15 OR #16 OR #17 OR #18 OR #19 OR #20 OR #21 OR #22 OR #23 OR #24 OR #25 OR #26 OR #27 OR #28 OR #29 OR #30 OR #31 OR #32 OR #33 OR #34 OR #35 OR #36 OR #37 OR #38 OR #39 OR #40 OR #41 OR #42 OR #43 OR #44 OR #45 OR #46 OR #47 OR #48 OR #49 OR #50 OR #51 OR #52 OR #53 OR #54 OR #55 OR #56 OR #57 OR #58 OR #59 OR #60 OR #61 OR #62 OR #63 OR #64 OR #65 OR #66 OR #67 OR #68 OR #69 OR #70 OR #71 OR #72 OR #73 OR #74 OR #75 OR #76 OR #77 OR #78 OR #79 OR #80 OR #81 OR #82 OR #83 OR #84 OR #85 OR #86 OR #87 OR #88 OR #89 OR #90 OR #91 OR #92 OR #93 OR #94 OR #95 OR #96 OR #97 OR #98 OR #99 OR #100 OR #101 OR #102 OR #103 OR #104 OR #105 OR #106 OR #107 OR #108 OR #109 OR #110 OR #111 OR

#112 OR #113 OR #114 OR #115 OR #116 OR #117 OR #118 OR #119 OR #120 OR #121 OR #122 OR #123 OR #124 OR #125 OR #126 OR #127 OR #128 OR #129 OR #130 OR #131 OR #132 OR #133

#135 Gram negative bacteria
#136 Enterobacteriaceae
#137 Enterobacterales
#138 Escherichia coli
#139 Klebsiella
#140 Enterobacter
#141 Proteus
#142 Serratia
#143 #135 OR #136 #OR #137 OR #138 OR #139 OR #140 OR #141 OR #142
#144 #7 AND #10 AND #134 AND #143

Table S1. Summary of the clinical studies published from inception to March 20222 that report the VF/VGs in GN neonatal sepsis.

Author, Year	Country/Center	Study Period	Study Design	Subjects	Study Sample	Analyses	Quality of Reporting #
1.Bryan K. Cole 2019 [1]	United States	Oct 2006–May 2016	Cohort study	Infants <90 day	43	1.Association VFs- neonatal mortality 2. Comparison of VF profile in EOS vs. LOS	52.2
2.Laura Folgori, 2021 [2]	United Kingdom, Estonia, Greece, Italy, Lithuania Spain	2010–2015	Cohort study	Infants < 90 days	87	1.Association VFs- neonatal mortality	57.6
3.Kathryn M Thomson, 2021 BARNARDS [3]	Bangladesh, Ethiopia, India, Nigeria, Pakistan, Rwanda, and South Africa	Nov 2005–Feb 2018	Prospective cohort study	Infants aged 0-60 days	70	1.Association VFs- neonatal mortality 2. Comparison of VF profile in EOS vs. LOS	71.7
4.Kirsty Sands, 2021 BARNARDS [4]	Bangladesh, Ethiopia, India, Nigeria, Pakistan, Rwanda, and South Africa	Nov 2015–Dec 2017	Prospective cohort study	Infants aged 0-60 days	258	1.Association VFs- neonatal mortality	78.3
5.Timo K Korhonen, 1985 [5]	Finland	1977–1981	Cross-sectional Multicentric	Neonates < 22 days	45	1.Comparison between bacteriemic strains vs. non-invasive strains 2. VF profile in neonatal sepsis vs. older children and adults' bacteremia	14.1
6.Stéphane Bonacorsi, 2006 [6]	France	Jan 1992–Dec 2022	Retrospective cohort	Infants < 90 days	32	1.Comparison between bacteriemic strains vs. non-invasive strains	27.3
7.Emma Saez-Lòpez, BS, 2017 [7]	Spain	April 2013–Oct 2013	Outbreak report	Neonates*		1.Comparison between bacteriemic	40.6

						strains vs. non-invasive strains	
8.Anja Siitonen 1993 [8]	Finland	1985–1989	Cross-sectional<br><br>Multicentric (23 hospitals)	Neonates <7 days	43	1.Comparison between bacteriemic strains vs. non-invasive strains<br><br>2.VF profile in neonatal sepsis vs. older children and adults' bacteremia	22.8
9.Stéphane Watt 2003 [9]	France Multicenter (12 hospitals)	1989–1997	Cross-sectional	Neonates*	29	1.Comparison between bacteriemic strains vs. non-invasive strains	20.6
10. R Tapader, 2014 [10]	India Multicenter	2000–2011	Cross-sectional	Neonates*	70	1.Comparison between bacteriemic strains vs. non-invasive strains	16.3
11. A Chmielarczyk, 2013 [11]	Poland	-	Abstract (conference proceeding)<br><br>Multicentric (6 hospitals)	Neonates*	24	1.Comparison between bacteriemic strains vs. non-invasive strains	18
12. K. Huik [12]	Estonia	Aug 2006–Dec 2007	Abstract (conference proceeding)<br><br>Multicentric (2 hospitals)	Neonates*	5	1.Comparison between bacteriemic strains vs. non-invasive strains	8.7
13. Susan W. Cook, 2001 [13]	United States	-	Cross sectional	Neonates*	45	1.Comparison between bacteriemic strains vs. non-invasive strains	29.3
14. Subhasree	India	2011	Point prevale	Neonates*	4	1.Comparison between	54.1

Roy, 2011 [14]			nce survey			bacteriemic strains vs. non-invasive strains	
15 Charles Burdet, 2014 [15]	France	Jan 2005–Nov 2007	Observational Prospective cohort<br>Multicentric (15 hospitals)	Infants <90 days	43	1.VF profile in neonatal sepsis vs. older children and adults' bacteremia<br>2.Portal of entry	43.5
16 Noemí Palma, 2016 [16]	Perù	2008–2011	Prospective cohort<br>Multicentric (12 hospitals)	Infants ≤3 months	49	1.VF profile in neonatal sepsis vs. older children and adults bacteremia<br>2. Comparison of VF profile in EOS vs. LOS	30.8
17. W.R. McCbe, 1978 [17]	United States	-	Cross-sectional<br>Multicentric (2 hospitlas)	Neonates*	46	1.VF profile in neonatal sepsis vs. older children and adults' bacteremia	20.2
18.S. M. Soto, 2008 [18]	Spain	1987–2006	Retrospective cohort	Neonates < 28 days	47	1.Comparison of VF profile in EOS vs. LOS	21.7
19. Farah Mahjoub-Messai, 2011 [19]	France	1999–2009	Prospective cohort	Infants < 90 days	49	1.Comparison of VF profile in EOS vs. LOS<br>2.Portal of entry	34.8
20. SJ Weissman, 2015 [20]	United States	2008–2009	Prospective cohort<br>Multicentric (16 hospitals)	Neonates *	28	1.Virulence profile in term vs. preterm neonates	45.6

* Not specified. # Percentage of STROBE-NI items adequately reported.

References

1. Cole, B.K.; Ilikj, M.; McCloskey, C.B.; Chavez-Bueno, S. Antibiotic resistance and molecular characterization of bacteremia Escherichia coli isolates from newborns in the United States. *PLoS ONE* **2019**, *14*, e0219352. Available online: http://ovidsp.ovid.com/ovidweb.cgi?T=JS&PAGE=reference&D=med16&NEWS=N&AN=31276562 (accessed on 16 June 2022
2. Thomson, K.M.; Dyer, C.; Liu, F.; Sands, K.; Portal, E.; Carvalho, M.J.; Barrell, M.; Boostrom, I.; Dunachie, S.; Farzana, R.; et al. Effects of antibiotic resistance, drug target attainment, bacterial pathogenicity and virulence, and antibiotic access and affordability on outcomes in neonatal sepsis: An international microbiology and drug evaluation prospective substudy (BARNARDS). *Lancet Infect. Dis.* **2021**, *21*, 1677–1688. Available online: https://www.embase.com/search/results?subaction=viewrecord&id=L2015715280&from=export (accessed on 16 June 2022).
3. Folgori, L.; Di Carlo, D.; Comandatore, F.; Piazza, A.; Witney, A.; Bresesti, I.; Hsia, Y.; Laing, K.; Monahan, I.; Bielicki, J.; et al. Antibiotic susceptibility, virulome, and clinical outcomes in european infants with bloodstream infections caused by enterobacterales. *Antibiotics* **2021**, *10*, 706. Available online: https://www.embase.com/search/results?subaction=viewrecord&id=L2007601246&from=export (accessed on 16 June 2022).
4. Lam, M.M.C.; Wick, R.R.; Watts, S.C.; Cerdeira, L.T.; Wyres, K.L.; Holt, K.E. A genomic surveillance framework and genotyping tool for Klebsiella pneumoniae and its related species complex. *Nat. Commun.* **2021**, *12*, 785. https://doi.org/10.1038/s41467-021-24448-3.
5. Korhonen, T.K.; Valtonen, M.V.; Parkkinen, J.; Väisänen-Rhen, V.; Finne, J.; Orskov, F.; Orskov, I.; Svenson, S.B.; Mäkelä, P.H. Serotypes, hemolysin production, and receptor recognition of Escherichia coli strains associated with neonatal sepsis and meningitis. *Infect. Immun.* **1985**, *48*, 486–491. Available online: http://ovidsp.ovid.com/ovidweb.cgi?T=JS&PAGE=reference&D=med2&NEWS=N&AN=2580792 (accessed on 17 June 2022).
6. Bonacorsi, S.; Houdouin, V.; Mariani-Kurkdjian, P.; Mahjoub-Messai, F.; Bingen, E. Comparative prevalence of virulence factors in Escherichia coli causing urinary tract infection in male infants with and without bacteremia. *J. Clin. Microbiol.* **2006**, *44*, 1156–1158. Available online: http://ovidsp.ovid.com/ovidweb.cgi?T=JS&PAGE=reference&D=med6&NEWS=N&AN=16517919 (accessed on 16 June 2022).
7. Sáez-López, E.; Bosch, J.; Salvia, M.D.; Orth, D.F.; Cepas, V.; Ferrer-Navarro, M.; Figueras-Aloy, J.; Vila, J.; Soto, S.M. Outbreak Caused by Escherichia coli O18:K1:H7 Sequence Type 95 in a Neonatal Intensive Care Unit in Barcelona, Spain. *Pediatr. Infect. Dis. J.* **2017**, *36*, 1079–1086. Available online: https://www.embase.com/search/results?subaction=viewrecord&id=L616990558&from=export (accessed on 16 June 2022).
8. Siitonen, A.; Takala, A.; Ratiner, Y.A.; Pere, A.; Makela, P.H. Invasive Escherichia coli infections in children: Bacterial characteristics in different age groups and clinical entities. *Pediatr. Infect. Dis. J.* **1993**, *12*, 606–612. Available online: http://ovidsp.ovid.com/ovidweb.cgi?T=JS&PAGE=reference&D=med3&NEWS=N&AN=8102199 (accessed on 16 June 2022).
9. Watt, S.; Lanotte, P.; Mereghetti, L.; Moulin-Schouleur, M.; Picard, B.; Quentin, R. Escherichia coli strains from pregnant women and neonates: Intraspecies genetic distribution and prevalence of virulence factors. *J. Clin. Microbiol.* **2003**, *41*, 1929–1935. Available online: http://ovidsp.ovid.com/ovidweb.cgi?T=JS&PAGE=reference&D=med5&NEWS=N&AN=12734229 (accessed on 17 June 2022).
10. Tapader, R.; Chatterjee, S.; Singh, A.K.; Dayma, P.; Haldar, S.; Pal, A.; Basu, S. The high prevalence of serine protease autotransporters of Enterobacteriaceae (SPATEs) in Escherichia coli causing neonatal septicemia. *Eur. J. Clin. Microbiol. Infect. Dis.* **2014**, *33*, 2015–2024. Available online: http://ovidsp.ovid.com/ovidweb.cgi?T=JS&PAGE=reference&D=med11&NEWS=N&AN=24924922 (accessed on 17 June 2022).
11. Chmielarczyk, A.; Pobiega, M.; Romaniszyn, D.; Wojkowska-Mach, J.; Heczko, P. Analysis of resistance, virulence, and phylogenetic groups of Escherichia coli strains isolated from very-low birth weight infants in Poland. *Antimicrob. Resist. Infect. Control* **2013**, *2*, P76. Available online: https://www.embase.com/search/results?subaction=viewrecord&id=L72036316&from=export (accessed on 16 June 2022).
12. Huik, K.; Parm, Ü.; Denks, K.; Pauskar, M.; Avi, R.; Metsvaht, T.; Ilmoja, M.; Lutsar, I.; Development of neonatal sepsis is not dependent on the presence of magA, k2A, rmpA and kfu genes in Klebsiella pneumoniae. *Clin. Microbiol. Infect.* **2010**, *16*, S589. Available online: https://www.embase.com/search/results?subaction=viewrecord&id=L70196586&from=export (accessed on 16 June 2022).
13. Cook, S.W.; Hammill, H.A.; Hull, R.A. Virulence factors of Escherichia coli isolated from female reproductive tract infections and neonatal sepsis. *Infect. Dis. Obstet. Gynecol.* **2001**, *9*, 203–207. Available online: http://ovidsp.ovid.com/ovidweb.cgi?T=JS&PAGE=reference&D=med4&NEWS=N&AN=11916176 (accessed on 16 June 2022).
14. Roy, S.; Singh, A.K.; Viswanathan, R.; Nandy, R.K.; Basu, S. Transmission of imipenem resistance determinants during the course of an outbreak of NDM-1 Escherichia coli in a sick newborn care unit. *J. Antimicrob. Chemother.* **2011**, *66*, 2773–2780. Available online: http://ovidsp.ovid.com/ovidweb.cgi?T=JS&PAGE=reference&D=med8&NEWS=N&AN=21930573 (accessed on 18 June 2022).
15. Burdet, C.; Clermont, O.; Bonacorsi, S.; Laouénan, C.; Bingen, E.; Aujard, Y.; Mentré, F.; Lefort, A.; Denamur, E.; COLIBAFI Group. Escherichia coli bacteremia in children: Age and portal of entry are the main predictors of severity. *Pediatr. Infect. Dis. J.* **2014**, *33*, 872–879. Available online: http://ovidsp.ovid.com/ovidweb.cgi?T=JS&PAGE=reference&D=med11&NEWS=N&AN=25222308 (accessed on 17 June 2022).

16. Palma, N.; Gomes, C.; Riveros, M.; García, W.; Martinez-Puchol, S.; Ruiz-Roldán, L.; Mateu, J.; Garcia, C.; Jacobs, J.; Ochoa, T.; et al. Virulence factors profiles and ESBL production in Escherichia coli causing bacteremia in Peruvian children. *Diagn. Microbiol. Infect. Dis.* **2016**, *86*, 70–75. Available online: http://ovidsp.ovid.com/ovidweb.cgi?T=JS&PAGE=reference&D=med13&NEWS=N&AN=27345125 (accessed on 17 June 2022).
17. McCabe, W.R.; Kaijser, B.; Olling, S. Escherichia coli in bacteremia: K and O antigens and serum sensitivity of strains from adults and neonates. *J. Infect. Dis.* **1978**, *138*, 33–41. Available online: https://www.embase.com/search/results?subaction=viewrecord&id=L8399825&from=export (accessed on 16 June 2022).
18. Soto, S.M.; Bosch, J.; Jimenez de Anta, M.T.; Vila, J. Comparative study of virulence traits of Escherichia coli clinical isolates causing early and late neonatal sepsis. *J. Clin. Microbiol.* **2008**, *46*, 1123–1125. Available online: http://ovidsp.ovid.com/ovidweb.cgi?T=JS&PAGE=reference&D=med7&NEWS=N&AN=18160454 (accessed on 16 June 2022).
19. Mahjoub-Messai, F.; Bidet, P.; Caro, V.; Diancourt, L.; Biran, V.; Aujard, Y.; Bingen, E.; Bonacorsi, S. Escherichia coli isolates causing bacteremia via gut translocation and urinary tract infection in young infants exhibit different virulence genotypes. *J. Infect. Dis.* **2011**, *203*, 1844–1849. Available online: http://ovidsp.ovid.com/ovidweb.cgi?T=JS&PAGE=reference&D=med8&NEWS=N&AN=21550999 (accessed on 16 June 2022).
20. Weissman, S.J.; Hansen, N.I.; Zaterka-Baxter, K.; Higgins, R.D.; Stoll, B.J. Emergence of Antibiotic Resistance-Associated Clones Among Escherichia coli Recovered From Newborns With Early-Onset Sepsis and Meningitis in the United States, 2008–2009. *J. Pediatr. Infect. Dis. Soc.* **2016**, *5*, 269–276. Available online: http://ovidsp.ovid.com/ovidweb.cgi?T=JS&PAGE=reference&D=med13&NEWS=N&AN=26407251 (accessed on 19 June 2022).
